# Draft Genomes of Heterogeneous Vancomycin-Intermediate *Staphylococcus aureus* Strain MM66 and MM66 Derivatives with Altered Vancomycin Resistance Levels

**DOI:** 10.1128/genomeA.00688-14

**Published:** 2014-07-10

**Authors:** Stephanie A. Matyi, Thiruvarangan Ramaraj, Anitha Sundararajan, Ingrid E. Lindquist, Nicolas P. Devitt, Faye D. Schilkey, Reena Lamichhane-Khadka, Peter R. Hoyt, Joann Mudge, John E. Gustafson

**Affiliations:** a Department of Biochemistry and Molecular Biology, Oklahoma State University, Stillwater, Oklahoma, USA; b National Center for Genome Resources (NCGR), Santa Fe, New Mexico, USA; c Department of Biology, New Mexico State University, Las Cruces, New Mexico, USA; d Department of Biology, Saint Mary’s College, Notre Dame, Indiana, USA

## Abstract

The draft genomes of heterogeneous vancomycin-intermediate *Staphylococcus aureus* (hVISA) strain MM66 and MM66 isolates demonstrating altered vancomycin resistance levels were produced in an effort to provide information on mutations contributing to the vancomycin resistance levels observed in these strains*.*

## GENOME ANNOUNCEMENT

The use of vancomycin for the treatment of methicillin-resistant *Staphylococcus aureus* (MRSA) infections (1, 2) has been challenged by the emergence of vancomycin-intermediate (3) and -resistant (4, 5) *S. aureus* (VISA and VRSA, respectively). We reported on a heterogeneous vancomycin-intermediate *S. aureus* (hVISA) strain (MM66) isolated from a hospital in Las Cruces, New Mexico, that when grown in the presence of vancomycin gave rise to stable VISA subpopulations (6). In order to provide insight on the MM66 hVISA mechanism, we have completed and compared the draft genomes of MM66, an MM66 VISA (MM66-4) (6), and a reduced vancomycin-intermediate (RVI) MM66 isolate (MM66RVI-4).

MM66RVI-4 was obtained by passaging MM66 in Luria broth (LB) and selecting for colonies unable to grow on LB agar containing 1 µg/ml vancomycin. The DNA of all strains was isolated from overnight cultures (37° C, 200 rpm) grown in Mueller-Hinton broth (MHB). Draft genomes of MM66 and MM66RVI-4 were produced with the Roche 454 GS (Junior) pyrosequencing platform and assembled using the Roche GS *de novo* assembler (v. 2.7). Libraries of MM66 and MM66-4 were constructed with the phusion-based Illumina genomic DNA library preparation protocol and sequenced using Illumina genome analyzer II 90-bp paired-end reads. *De novo* assembly was generated using filtered sequence reads and the ABySS assembler (v. 1.3.7) (7). All genome sequences were uploaded to the RAST server for annotation (8).

The vancomycin Etest MIC (6) of MM66RVI-4 and MM66 were 2 µg/ml and 3 µg/ml, respectively. MM66RVI-4 also demonstrated decreased distances grown on 0 to 3 µg/ml vancomycin (41.6 mm ± 3) and 0 to 3 µg/ml teicoplanin (16 mm ± 3) gradients (9) compared to MM66 (61.6 mm ± 4, and 30.6 mm ± 4, respectively (*n* = 3; *P* ≤ 0.05). In addition, MM66RVI-4 did not grow as well in MHB containing 2.5 µg/ml vancomycin and demonstrated reduced cell survival in vancomycin resistance population analysis (9) (0 to 3.5 µg/ml) performed with MH agar. Furthermore, whereas MM66 grew on a 0 to 175 µg/ml oxacillin gradient (84.0 mm ± 2), MM66RVI-4 grew only on a 0 to 0.5 µg/ml gradient (73 mm ± 5).

Draft genome information for all strains is summarized in Table 1. The number of RAST predicted protein-coding sequences were 2,684 (454) and 2,858 (Illumina) for MM66 and 2,563 and 2,814 for MM66RVI-4 and MM66-4, respectively. All strains sequenced were multilocus sequence type 5 (ST-5) and staphylococcal cassette chromosome *mec* type II (SCC*mec*II) (10, 11). The isolation ST-5/SCC*mec*II strains in the Las Cruces area have been previously reported (12, 13). The loss of oxacillin resistance in MM66RVI-4 is corroborated by an ~46.5-kb deletion of SCC*mec*II that remains in MM66 and MM66-4. Selection for VISA in the laboratory can also lead to SCC*mec* loss (14, 15). Mutations in genes encoding the two-component GraSR system are thought to support the VISA mechanism (16–19) and all MM66 derivatives harbored the same mutation in *graS* (S270N). In addition, mutations within *apt* and *yycG* of MM66-4 (20) were confirmed.

**TABLE 1:** *Staphylococcus aureus* genome assembly and accession numbers

Strain	Sequence coverage (%)	No. of contigs (bp)	*N*_50_ (Mbp)	Genome length (bp)	GC content (%)	Accession no.
MM66	30.1^*a*^	114 (>200)	0.17	2,834,320	32.9	JMBT00000000
MM66	348^*b*^	90 (>1,000)	0.09	3,002,171	33.0	CCCM000000000
MM66RVI-4	36.6^*a*^	197 (>200)	0.21	2,732,996	33.0	JMBU00000000
MM66-4	612^*b*^	73 (>1,000)	0.15	2,940,194	32.8	CCCI000000000

a 454 sequence coverage.

b Illumina sequence coverage.

### Nucleotide sequence accession numbers.

These whole-genome shotgun projects have been deposited at DDBJ/EMBL/GenBank under the accession numbers JMBT00000000, CCCM000000000, JMBU00000000, and CCCI000000000.
